# Unruptured ovarian pregnancy following *in-vitro* fertilization: Missed diagnosis followed by successful laparoscopic management

**DOI:** 10.4103/0974-1208.39596

**Published:** 2008

**Authors:** Narvekar SA, VijayKumar PK, Shetty N, Gupta N, Ashwini GB, Rao KA

**Affiliations:** Bangalore Assisted Conception Center, #6/7, Kumara Krupa, High Grounds, Bangalore - 560 001, India

**Keywords:** *In-vitro* fertilization, laparoscopy, ovarian pregnancy

## Abstract

Ovarian pregnancy after *in-vitro* fertilization is rare and can be easily missed unless there is a high index of suspicion. Here we present such a case which was missed initially but was later successfully managed laparoscopically.

Although the incidence of ectopic pregnancy is on the rise, ovarian pregnancy after *in-vitro* fertilization and embryo transfer (IVF-ET) is rare. Ovarian pregnancy accounts for 0.5-3% of all ectopic pregnancies[1] and the incidence of ovarian pregnancy after IVF has been reported to be 0.3%.[2] Here we report a case of unruptured ovarian pregnancy, following IVF-ET which was missed initially but later successfully managed laparoscopically.

## CASE REPORT

The patient was 35-year-old with secondary infertility of 4 years. She had four natural conceptions, the first three of which were spontaneous first trimester abortions and the fourth a ruptured ectopic for which she underwent a laparotomy with right total salpingectomy.

She had regular cycles with normal hormonal profile. Her husband was detected to have mild asthenospermia. After four unsuccessful attempts at ovulation induction with intrauterine insemination, controlled ovarian hyperstimulation with IVF was planned for her.

After luteal phase downregulation with leuprolide acetate (Lupride® Sun Pharmaceutical Halol, Gujarat India Ltd) and stimulation with recombinant follicle stimulating hormone (Recagon® Organon, Ireland Pvt Ltd), five oocytes were retrieved from the right ovary. The left ovary did not yield any follicles. Three eight-cell stage embryos were transferred on day 3 under ultrasound guidance about 1.5 cm away from the fundus using Edward-Wallace catheter (Simcare, West Sussex, UK) with 20 µl of culture fluid.

On day 14 after ET, her serum βhCG was 617.9 IU/l and on day 18, it increased to 4779.3 IU/l at which point the transvaginal scan revealed a thickened endometrium of 13 mm with multiple hemorrhagic corpora lutea in the right adnexa and small follicles in the left ovary. But we could not demonstrate any intra/extrauterine gestational sac. We decided to keep her under close observation for 48 h as she was asymptomatic and the initial rise in βhCG appeared adequate if multiple intrauterine gestations were considered. On day 20 after ET, the βHCG increased to 8350 IU/l. This time again, we could not demonstrate a gestational sac on scan. Hence we decided to perform a laparoscopy which revealed a bulky uterus with absent right tube and multiple hemorrhagic corpora lutea in the right ovary which was adhered to the lateral pelvic wall. The left tube and ovary were normal. There was 100 ml of straw colored fluid in the pouch of douglas (POD). Pregnancy could not be visualized elsewhere in the abdomen.

The patient was subsequently followed up with βhCG which showed a steady rise from 11,719 IU/l on 24^th^ day to 22,443 on 28^th^ day and then plateaued to 26,529 on day 30 of embryo transfer. At this point, a transvaginal scan was performed which revealed a gestational sac with a thick echogenic rim and a live fetus corresponding to 7 weeks within the substance of the right ovary. The patient now complained of bleeding PV, but no abdominal pain.

**Figure 1:**
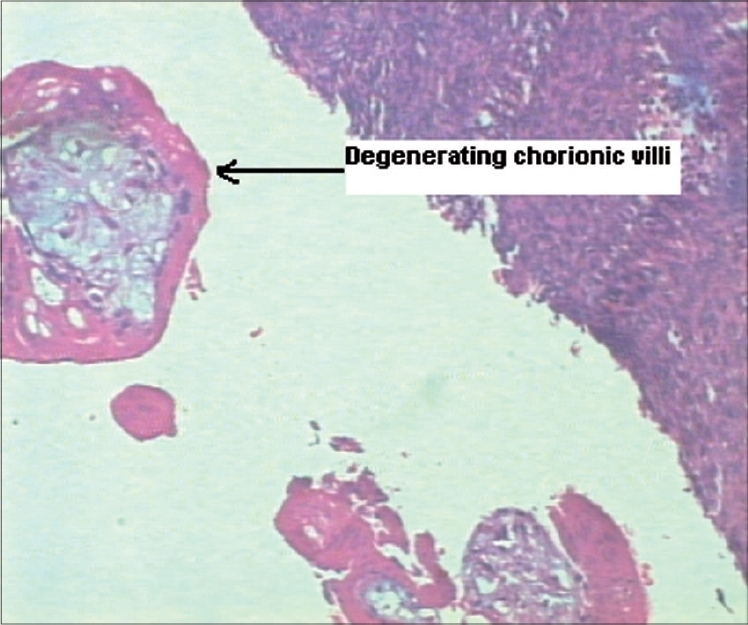
Microscopic view showing degenerating chorionic villi (45×)

**Figure 2:**
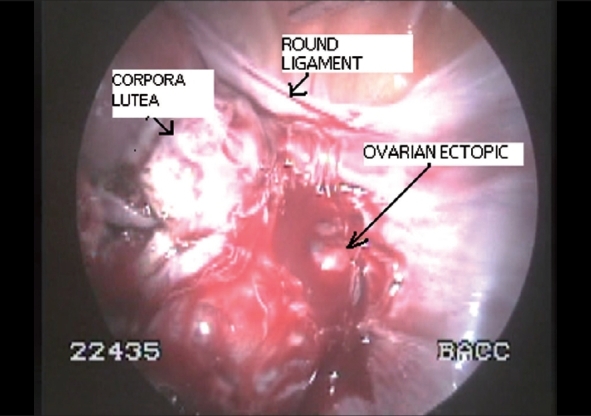
Laparoscopic view showing multiple hemorrhagic corporalutea with the ovarian pregnancy buried within

We performed laparoscopy for the second time and excised the suspicious cysts and visually inspected each one of them but failed to identify any chorionic/fetal tissue. We then performed vaginal scan after filling the pelvis with saline to locate the pregnancy. The gestational sac was deeply seated within the right ovary which was adhered to the lateral pelvic wall close to the iliac vessels making selective excision of the sac difficult hence an oopherectomy was performed [Figure 1]. Histopathological examination confirmed the diagnosis of ovarian pregnancy. The patient had an uneventful recovery and is planned for IVF [Figure 2].

## DISCUSSION

Ovarian pregnancy following IVF is rare and to our knowledge only 13 cases have been reported so far.[2,3]

The Spiegelberg's criteria[4] for an ovarian pregnancy include: (1) fallopian tubes, including fimbria, must be intact and separate from the ovary, (2) the pregnancy must occupy the normal position of the ovary, (3) the ovary must be attached to the uterus through the uteroovarian ligament, and (4) there must be ovarian tissue attached to the pregnancy in the specimen.

Our case did not meet the first criterion, as she had undergone an ipsilateral salpingectomy, but satisfied the rest.

This form of implantation could be the result of reverse migration of the embryo as a result of deep deposition of the embryo into the uterine cavity[5] or the use of large volume of culture fluid during transfer.[6] The presence of tubal pathology and pelvic inflammatory disease could also be the predisposing factors as with tubal pregnancies.

Ovarian pregnancy on sonography is typically described as a wide echogenic ring with a small internal echolucent area on the surface or within the substance of the ovary, the echogenicity of the ring usually being greater than the ovary.[7] A high degree of suspicion is needed, particularly in stimulated ovaries, wherein it can easily be mistaken for hemorrhagic corpora lutea. Doppler ultrasonography seems to offer little additional diagnostic value due to the high vascularity of the ovary.[7] In our case, we could identify the ovarian pregnancy only after the fetal pole appeared. The lesson that we learnt is that a thorough search by dissecting away the corpus luteum cysts at the time of laparoscopy should be done, to avoid missing an ovarian ectopic. An intraoperative vaginal scan aids in localization of the gestational sac within the ovary. Selective excision the gestational sac is the treatment of choice.[8]

There are few reports of successful use of systemic methotrexate in ovarian pregnancy.[9,10] Successful treatment of ovarian pregnancy with 50-mg methotrexate injected into the ectopic sac at laparoscopy has also been reported.[11] Because of the limited literature available, unlike in tubal pregnancies, the factors predicting success with systemic methotrexate in ovarian pregnancy are not well defined.

We believe that laparoscopy remains the gold standard for diagnosis as well as treatment of ovarian pregnancy.
